# Relationship between fracture-relevant parameters of thoracolumbar burst fractures and the reduction of intra-canal fracture fragment

**DOI:** 10.1186/s13018-015-0260-2

**Published:** 2015-08-27

**Authors:** Ye Peng, Licheng Zhang, Tao Shi, Houchen Lv, Lihai Zhang, Peifu Tang

**Affiliations:** Department of Orthopedics, Chinese PLA General Hospital, No. 28 Fuxing Road, Beijing, 100853 People’s Republic of China

**Keywords:** Single thoracolumbar burst fracture, Posterior longitudinal ligament, Fracture reduction, Intra-canal fracture fragment

## Abstract

**Objective:**

Posterior longitudinal ligament reduction (PLLR) has been widely used for treatment of thoracolumbar burst fractures. However, there are no systemic studies assessing the influence of position parameters of intra-canal fracture fragment (IFF) itself on outcome of reduction. The aim of this study was to analyze the relationship between position parameters of IFF and the reduction efficacy of PLLR.

**Methods:**

Sixty-two patients (average age, 36.9 years) with single thoracolumbar burst fractures and intact posterior longitudinal ligaments were recruited. Patients were divided into reduced and unreduced groups based on IFF reduction situations by PLLR. Preoperative and intraoperative computed tomography (CT) were used to evaluate reduction and location parameters of IFF, such as position, width, height, inversion, and horizontal angle, ratio of width of IFF to the transverse diameter of vertebral canal (*R*_1_), and ratio of height of IFF to height of injured vertebrae (*R*_2_) before and after PLLR.

**Results:**

There were significant differences in width (*P* < 0.001), height (*P* = 0.0141; *R*_1_, *P* < 0.001), and *R*_2_ (*P* = 0.0045) between the two groups. When width of IFF was more than 75 % of transverse diameter of vertebral canal and height of IFF was more than 47 % of height of injured vertebrae, the IFF could not be reduced by PLLR.

**Conclusions:**

In patients with thoracolumbar burst fractures, IFF in apterium of the posterior longitudinal ligament cannot be reduced by PLLR. For thoracolumbar burst fractures that cover the posterior longitudinal ligament, the width and height of IFF are important parameters that influence reduction quality.

## Introduction

Each year, 13.3–45.9 of every 1,000,000 people suffer from spinal trauma. Ninety percent of fractures occur in the thoracolumbar spine, and thoracolumbar burst fractures account for 20 % of these. Among patients with thoracolumbar burst fractures, 50−60 % also experience neurologic deficit [1–6]. The major causes of spinal fractures are traffic accidents (43 %), falling from a significant height (25 %), and a violent incident (16.5 %) [7].

The spinal cord suffers both primary and secondary damage after acute spinal cord injury. However, it is difficult to avoid primary damage since it usually occurs very rapidly. Therefore, current therapeutic strategies for spinal cord injury (SCI) primarily focus on reducing the severity of secondary damage. Secondary mechanisms of injury encompass an array of perturbances and include variation of the local blood vessels [8, 9], electrolyte disturbance [10, 11], cellular apoptosis [12], and other miscellaneous processes. It has been shown that continuous mechanical compression of the neural structure by intra-canal fracture fragment is the main reason for secondary damage.

Spinal decompression is said to be more helpful to neurological recovery. And there are three surgical approaches to decompression: the anterior approach, posterior approach, and the combined approach. The anterior approach has generally been applied to patients who sustained severe communicated, reversed bony fragments, or kyphotic deformity [13]. However, this approach has obvious shortcomings: larger trauma, longer operation time, higher costs, and high incidence of complications such as bleeding, vascular injury, and pneumothorax. The posterior approach is the most familiar and widely accepted method to most spinal surgeons [14]. The approach ensures safe exposure of the operation field, and it is effective for correction of the kyphotic deformity, restoration of vertebral height, and nerve function. Other advantages include shorter operation time, less blood loss, reduced cost, and less postoperative complications.

In the 1980s, it was first reported that the external fixator through the posterior approach could transmit a longitudinal distraction force by tensing the PLL and helps to restore skeletal anatomy. At present, posterior operation based on internal fixation has become popular to achieve the reduction of intra-canal fracture fragment and kyphosis correction [15, 16]. However, not all intra-canal fracture fragments can be reduced using internal fixation through posterior approach. There are still some patients who would need intraoperative spinal canal decompression. Zausinger et al. [17] reported that pseudo-inversion occurred in intra-canal fracture fragment. This made it easy to mistake the posterior wall of intra-canal fracture fragment for the anterior wall on a computer tomography scan (CT). In fact, the image they reported was that the vertebral upper endplate concaved into the vertebral body [17]. Steudel et al. [18] confirmed the existence of fracture fragment inversion on images and reported that these inversions might become obstacles for intra-canal fracture fragment reduction during treatment. Although Mueller suggested that “swing-like” fracture fragments could not be reduced by posterior longitudinal ligament reconstruction, the fracture fragments were not described in more details [19].

To the best of my knowledge, there are no systemic studies assessing the influence of position, size, and location parameter of the fracture block itself on the reduction of fracture fragment. The aim of this study was to investigate the relationships between height, width, sagittal inversion angle, and horizontal rotation angle of the intra-canal fracture fragment, the ratio of the height of intra-canal fracture fragment to the height of posterior wall of the injured vertebrae, and the ratio of the width of intra-canal fracture fragment to the width of the injured vertebrae during fracture fragment reduction in thoracolumbar burst fractures.

## Materials and methods

### Experimental instruments

Sensation Open 40 model (Germany, Siemens AG) was used for intraoperative CT. The imaging workstation was from Siemens AG, and Syngo Image processing software (Siemens AG) was used for image processing. A universal spine system (USS) internal fixation system for spinal injury (Sindis) was selected for the internal fixation operations.

### Subjects

All patients included in the study underwent MRI before surgery, and the integrity of the PLL was assessed by preoperative MRI scans. Patients with single thoracolumbar burst fractures caused by trauma and intact posterior longitudinal ligaments who were hospitalized between January 2009 and December 2011 were enrolled in the study. The exclusion criteria included multiple vertebral fractures, osteoporotic vertebral fracture, vertebra metastases, spondylolisthesis, ankylosing spondylitis, and osteoarthritis of the hip or knee. All investigations were carried out in accordance with the ethical guidelines and were approved by the Institutional Ethical Review Committee of Chinese PLA General Hospital.

### Operations

**Fig. 1:**
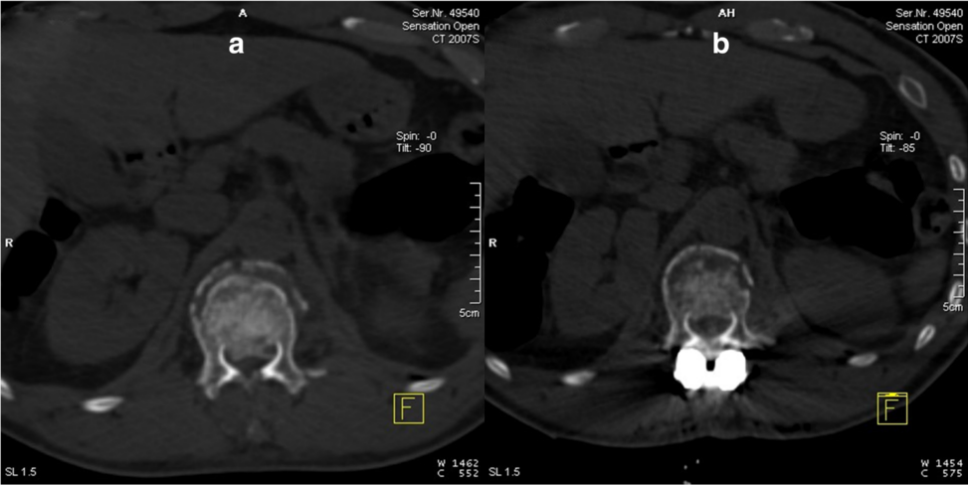
**a** CT picture of intra-canal fracture fragment before PLLR. **b** CT picture of intra-canal fracture fragment after PLLR

Patients were placed in the prone position, and their chests and hypogastria were raised using cushions. The operation was then performed after general anesthesia. Pressure was applied to the posterior side of the injured ventral body. Simultaneously, traction was applied along the long axis of body; manipulative reduction was performed. During the operation, a pedicle screw was implanted, the front column was dilated, and then the rear column was dilated. Lordosation and distraction with the internal fixator lead to the restoration of height, kyphosis correction, and canal widening by the phenomenon of ligamentotaxis. Intra-canal fracture fragment reduction was evaluated by intraoperative CT, and the typical CT pictures of intra-canal fracture fragment before and after posterior longitudinal ligament reduction (PLLR) were supplied (Fig. 1). If no reduction was observed, laminectomy was performed to depress the vertebral canal for intra-canal fracture fragment. The vertebral canal decompression range was determined based on intraoperative CT images.

### Measurement of bone block parameters

#### Position of the intra-canal fracture fragment

**Fig. 2:**
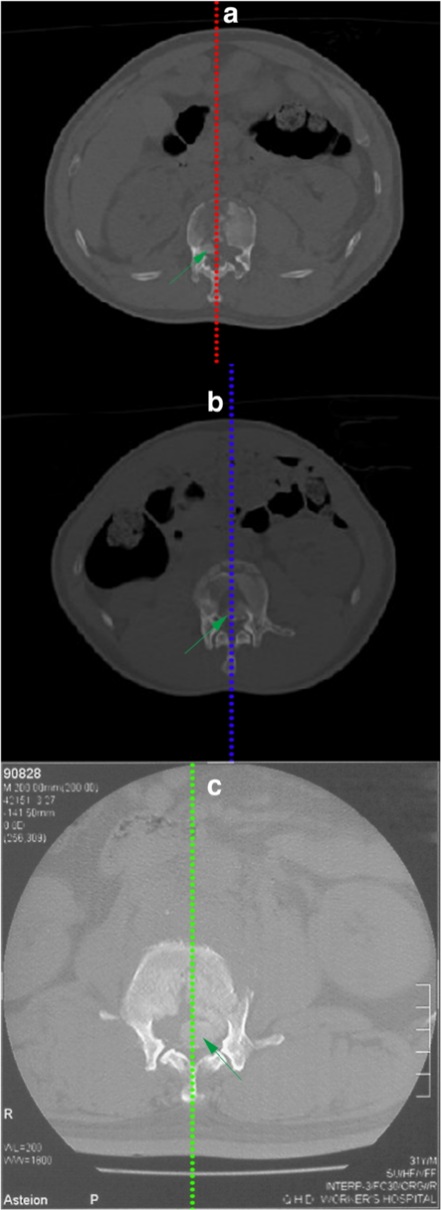
**a** Processus aboralis fracture block located in the left third of the posterior vertebral wall. **b** Processus aboralis fracture block located in the middle third of the posterior vertebral wall. **c** Processus aboralis fracture block located in the right third of the posterior vertebral wall

According to the preoperative CT results, the whole posterior vertebral wall was divided into trisections, and the type of intra-canal fracture fragment was defined as left (Fig. 2a), middle (Fig. 2b), or right sided (Fig. 2c).

#### Inversion angle of the intra-canal fracture fragment

**Fig. 3:**
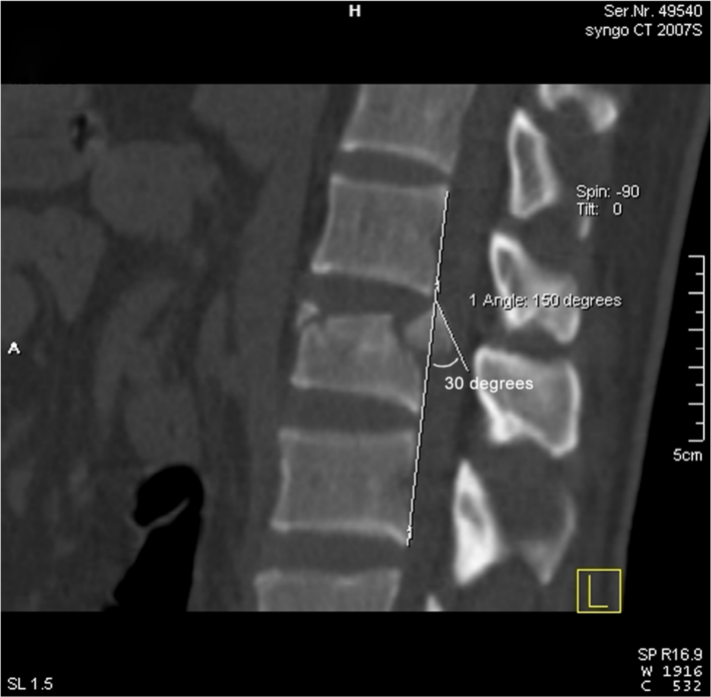
Measurement of the sagittal inversion angle of the processus aboralis fracture block; the angle in the example shown was 30°

In the median sagittal position, the connected line between the above and below adjacent posterior vertebral wall of the injured vertebral body was recorded as the standard posterior wall of the injured vertebral body. The intersection angle between the vertebral lateral bone cortex of the intra-canal fracture fragment and the standard posterior wall was recorded as the inversion angle (Fig. 3) [20].

#### Horizontal rotation angle of the intra-canal fracture fragment

**Fig. 4:**
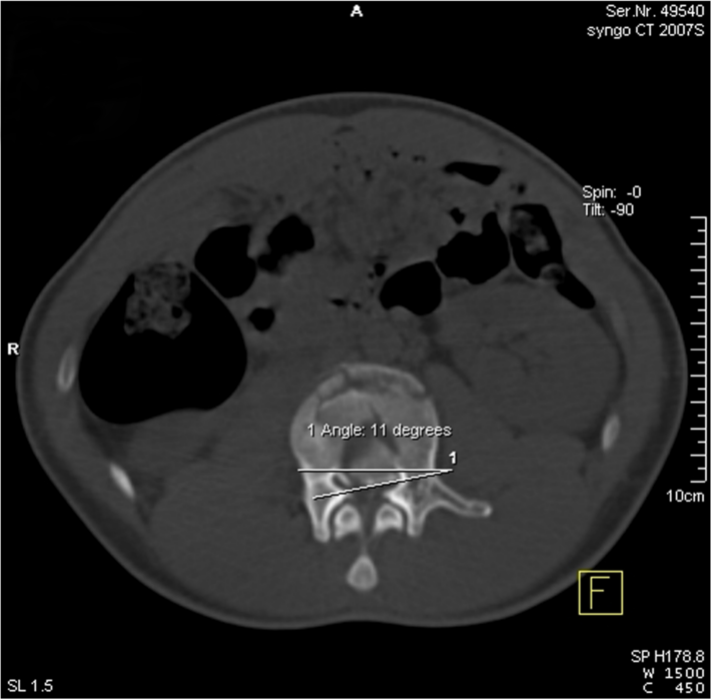
Measurement of the horizontal rotation angle of the processus aboralis fracture block; the angle in the example shown was 11°

The rotation angle referred to the angle of the intersection between the extension lines of the posterior vertebral wall and the intra-canal fracture fragment (Fig. 4) [21, 22].

#### Width and height of the intra-canal fracture fragment

**Fig. 5:**
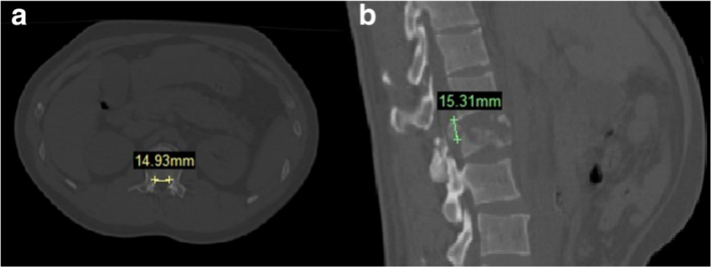
**a** Measurement of processus aboralis fracture block width; the width of the example shown was 14.93 mm. **b** Measurement of processus aboralis fracture block height; the height in the example shown was 15.31 mm

The width (Fig. 5a) and height (Fig. 5b) of the intra-canal fracture fragment was measured based on CT images.

#### The ratio (*R*_1_) of the width of the intra-canal fracture fragment to the transverse diameter of vertebral canal and the ratio (*R*_2_) of the height to the posterior wall of the injured vertebrae

**Fig. 6:**
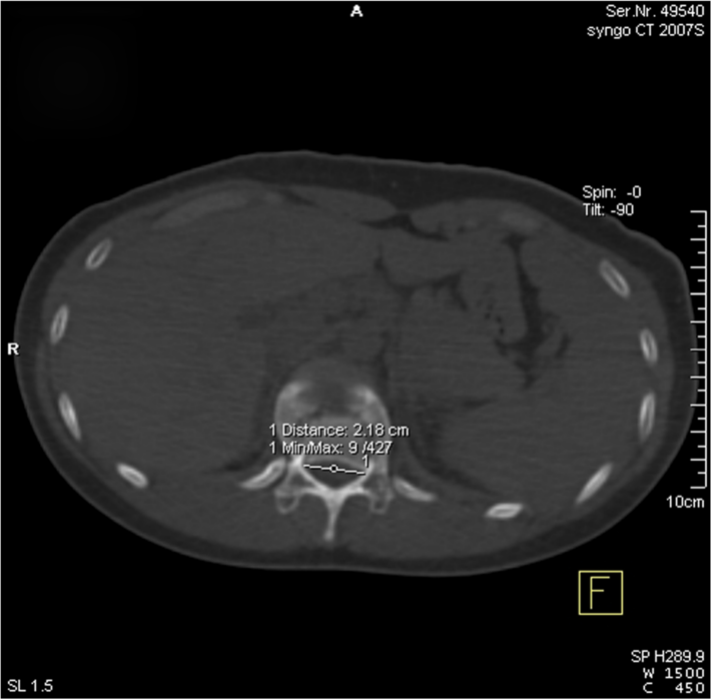
Measurements of the transverse diameter of the vertebral canal

We were unable to measure the transverse diameter of the vertebral canal of the injured vertebrae (Fig. 6) and the posterior vertebral wall height due to damage caused by the fracture. According to the method proposed by Hashimoto et al. [23], the average of the transverse diameter (ATD) of the above and below vertebral canal of the injured vertebrae was recorded as the normal transverse diameter of the vertebral canal of the injured segment. The average of posterior vertebral wall height (APT) of the above and below posterior vertebral wall height was recorded as the normal height of posterior vertebral wall of the injured segment. The width and height of the intra-canal fracture fragment were recorded as WF and HF. *R*_1_ and *R*_2_ can be calculated in the equation below:

$$ {R}_1 = \mathrm{W}\mathrm{P}/\mathrm{A}\mathrm{T}\mathrm{D} \times 100\ \%,\kern0.2em {R}_2 = \mathrm{H}\mathrm{P}/\mathrm{A}\mathrm{P}\mathrm{T} \times 100\ \% $$

### Grouping

Based on intraoperative CT scans, the intra-canal fracture fragment reduction situations were divided into reduced and unreduced status. A cross-statistical analysis was performed between reduction and non-reduction status with height, width, sagittal inversion angle, horizontal rotation angle of the intra-canal fracture fragment, and the median sagittal diameter compression ratio of the vertebral canal.

### Statistical methods

All data were analyzed using SPSS13.0 software. Quantitative data were expressed as $x\left(-\right)$ ± s, and qualitative data were expressed in terms of grade. Statistical analyses of the data were performed using non-paired *t* test. The difference was regarded statistically significant when *α* < 0.05.

## Results

### Patient data

A total of 97 patients had thoracolumbar burst fractures, of which 62 cases complied with the inclusion criteria (Table 1). Thirty-five patients were excluded, including 14 patients with two or more fractured vertebral bodies, 10 with fracture accompanied with diosmosis, and 11 patients with fracture accompanied with spondylolisthesis.

**Table 1:** Patient characteristics

Variables	Total
Male (*n*)	41
Female (*n*)	21
Mean age (years)	36.9
T_11_	5
T_12_	13
L_1_	26
L_2_	18
Cause of fracture	
Fall	36
Traffic accident	26

### Relationship between the position of the intra-canal fracture fragment and reduction

The bony fragment positions and reduction situations of 62 patients are shown in Table 2. Anatomical analysis revealed that the apterium of posterior longitudinal ligament existed at two sides of the posterior vertebra wall, and there was no posterior longitudinal ligament coverage. Therefore, intra-canal fracture fragments with fractures occurring on the left or right could not be reduced by posterior longitudinal ligament distraction. Only 55 patients with the bony fragment located in the region of posterior longitudinal ligament were selected to investigate the relationships between bone fragment parameters and reduction. Neurologic status was classified according to the scoring system of the American Spine Injury Association (ASIA); there were 20 patients in class A, 5 patients in class B, 8 patients in class C, 11 patients in class D, and the remaining 11 patients in class E. Of the 55 patients with bony fragment located in the middle region, 38 patients in whom the fragments were reduced by ligamentotaxis were included in the reduced group. In 17 patients, the fracture fragment in the spinal canal was not reduced, and these patients were included in the non-reduced group.

**Table 2:** The positions of the processus aboralis bony fragment and reduced and unreduced bony fragment according to intraoperative CT

Bony fragment position	*n*	Reduced	Unreduced
Left	3	0	3
Right	4	0	4
Middle	55	38	17

### Analysis of the intra-canal fracture fragment size and reduction

Table 3 shows the results of statistical analysis of fracture fragment measurement parameters and intra-operative bone block reduction situations in patients with intra-canal fracture fragment with fractures occurring in the middle. There was a significant difference in the width (*t* = 0.0141, *P* = 0.0141) and height (*t* = 2.5278, *P* = 0.0141) of the intra-canal fracture fragment between the reduction and non-reduction groups. There was also a significant difference in the ratio of the width of the intra-canal fracture fragment to the transverse diameter of the vertebral canal between the reduction and non-reduction groups (*t* = 4.695, *P* = 0.000016), as well as the ratio of the height of intra-canal fracture fragment height to that of the injured vertebrae (*t* = 2.9484, *P* = 0.0045). When the width of intra-canal fracture fragment was more than 75 % of the transverse diameter of the vertebral canal and the height of intra-canal fracture fragment was more than 47 % of that of the injured vertebrae, the intra-canal fracture fragment could not be reduced by posterior internal fixation and the posterior longitudinal ligament distraction reduction method.

**Table 3:** The processus aboralis bony fragment size and reduction

Parameters	Reduced	Unreduced	*P* value
	*n* = 38	*n* = 24	
Processus aboralis bony fragment width (mm)	13.57 ± 3.74	17.30 ± 4.27	0.0006
Processus aboralis bony fragment height (mm)	11.01 ± 3.36	13.02 ± 2.47	0.0141
Ratio of processus aboralis bony fragment width to transverse diameter of the vertebral canal (%)	54.3 ± 15.4	74.9 ± 18.9	0.000016
Ratio of the processus aboralis bony fragment height to the vertebral body height of the injured vertebrae (%)	39.1 ± 10.9	46.9 ± 8.8	0.0045

### Analysis of the spatial motion position of the intra-canal fracture fragment and reduction

Table 4 shows the results of statistical analysis of the spatial motion parameters of bone fragment and intraoperative bone fragment reduction in patients with intra-canal fracture fragment with fractures occurring in the middle. There was no significant difference in the sagittal inversion angle (*t* = 1.1695, *P* = 0.2468) and the horizontal rotation angle (*t* = 0.9138, *P* = 0.3645) of the intra-canal fracture fragment between the reduction and non-reduction groups.

**Table 4:** The spatial motion position of processus aboralis fracture block and reduction

Parameter	Reduced	Unreduced	*P* value
	*n* = 38	*n* = 24	
Sagittal inversion angle of the processus aboralis fracture block (°)	31.46 ± 16.70	36.17 ± 13.18	0.2468
Horizontal rotation angle of the processus aboralis fracture block (°)	2.81 ± 3.06	3.53 ± 2.96	0.0001
Median sagittal diameter compression ratio of the vertebral canal (%)	35.3 ± 18.8	59.6 ± 24.9	0.0001

## Discussion

Denis [24] investigated 412 patients with spinal fractures and proposed the spinal three-column theory to form a more detailed discussion on fractures using CT images. Among the thoracolumbar burst fractures defined using this theory, processus aboralis protrudes into the vertebral canal to form intra-canal fracture fragments. In the anatomical structure of the spine, the posterior vertebral wall contains the posterior longitudinal ligament, which clings to the posterior vertebral wall and is divided into two bundles (shallow and deep). The shallow layer extends from the foramen magnum downwards to the L3/4 intervertebral disc, with a width of 0.5−1 cm. The deep layer is segmental, with a width of 1 cm. In the annulus fibrosus, its fibers extend outward to become wide and fuse with the posterior annulus fibrosus wall and the posterior upper edge and vertebral pedicle periosteum of the next vertebral body. Therefore, the intra-canal fracture fragment of a thoracolumbar burst fracture is an anatomical structure closely related to the posterior longitudinal ligament, which plays an important role in treatment.

Many studies [19, 25–27] have been performed on the anatomical structures of the spine. It was suggested that intra-canal fracture fragment reduction is the result of both posterior longitudinal ligament reconstruction and tension caused by hyperextension of the posterior longitudinal ligament; these results are also effective in the process of non-operation and internal and external fixation treatment. However, some intra-canal fracture fragment cannot be reduced in the operation. The deep layer of the posterior longitudinal ligament is segmented (1 cm in with), and its fibers extend outwards at the annulus fibrosus and fuse with the next vertebral body. The posterior wall of this part cannot be covered by posterior longitudinal ligament completely. The results of the current study demonstrate that when intra-canal fracture fragment occurs on the left or right third of the vertebral body, intra-canal fracture fragment cannot be reduced by the posterior internal fixation and posterior longitudinal ligament distraction reduction method (Table 2). Combining the anatomical characteristics of the posterior longitudinal ligament, we found that there is no posterior longitudinal ligament coverage on the posterior side of the left or right third of the vertebral body (Fig. 6a, b). Therefore, intra-canal fracture fragment in this position cannot be reduced by the posterior longitudinal ligament reduction method. Similarly, the above analysis suggests that we could exclude intra-canal fracture fragment that occur on both sides from analyses of the relationships between vertebral body parameters and reduction. Therefore, it is possible to more accurately analyze the influences of bone block parameters on posterior longitudinal ligament reduction.

A biomechanical study by Oxland et al. [21] suggested that vertebral body motion is three-dimensional in space. Consistent with this, Manohar et al. [22] found that vertebral body rotated on the vertebral body level. Similarly, intra-canal fracture fragment of thoracolumbar burst fractures presented a three-dimensional motion and, particularly on the horizontal plane, exhibited rotation displacement. Therefore, the rotation angle of the posterior wall of intra-canal fracture fragment against the posterior vertebral wall is an important indicator for fracture block shape [21, 22]. However, there was no significant difference in sagittal inversion or horizontal rotation angles of the intra-canal fracture fragment between the reduction and non-reduction groups. This suggests that the rotation angles of the bone fragment in two directions of the vertebral body do not influence posterior longitudinal ligament reduction. There was a significant difference only in median sagittal diameter compression ratio of the vertebral canal between the reduction and non-reduction groups, suggesting that the size of posterior displacement in the horizontal direction influences the efficiency of posterior longitudinal ligament reduction. If the distance between intra-canal fracture fragment is more than a specific value, it will influence reduction, which might be associated with posterior longitudinal ligament integrity.

There was a significant difference in the bone fragment height and width between the reduction and non-reduction groups, suggesting that the size of intra-canal fracture fragment also influenced the restoration of bone fracture. Thoracolumbar vertebral size varies significantly according to gender and ethnicity. Moreover, the shape of intra-canal fracture fragment is irregular, and it is difficult to accurately measure their volume and area. Therefore, the ratios of the width of intra-canal fracture fragment to the transverse diameter of the vertebral canal and the height of intra-canal fracture fragment to the posterior wall height of the injured vertebrae were used to assess the fracture restoration in this study. Data suggested that when the width of intra-canal fracture fragment was more than 75 % of the transverse diameter of the vertebral canal and the height was more than 47 % of that of the injured vertebrae, the intra-canal fracture fragment could not be reduced by the posterior internal fixation and posterior longitudinal ligament distraction reduction method.

The posterior longitudinal ligament plays an important role in the reduction of small processus aboralis fracture blocks, but has no obvious effect in larger blocks. This suggests that the size of bone fragment is a main factor that determines the efficiency of posterior longitudinal ligament reduction. A study performed by Mueller et al. confirmed this hypothesis [19]. There are two possible reasons for a poor reduction efficacy in large trapezoid-like bone blocks: first, if the bone fragment is located in the posterior longitudinal ligament apterium on both sides of the posterior vertebral wall, it cannot be reduced by the posterior longitudinal ligament reduction method. Second, if the size of bone fragment and the distance of backward displacement are both large, the integrity of the posterior longitudinal ligament might be damaged. However, neither simple inversion nor lateral rotation influenced the efficiency of posterior longitudinal ligament reduction.

Although this study draws quantitative conclusions, the operation times included were limited to within 1–14 days of injury; therefore, the effects of operations performed after 14 days were not assessed. For patients who suffered thoracolumbar burst fractures more than 14 days, the condition of intra-canal fracture fragment is more complicated and more difficult to reduction. A concrete analysis should be performed carefully in clinical work. Although the correlations between the size and position of bone fragment and the reduction efficacy were observed, across analysis was not performed to assess the reduction efficacy and the combination of bone block space size and position.

## Conclusions

This study revealed that the position of the intra-canal fracture fragment is one of the major factors that determine whether the posterior internal fixation and posterior longitudinal ligament distraction reduction method can successfully reduce the intra-canal fracture fragment. When intra-canal fracture fragments are located in the apterium of posterior longitudinal ligament, they cannot be reduced. In contrast, in blocks located in areas covered by the posterior longitudinal ligament, the size of intra-canal fracture fragment becomes the main factor that influences reduction. When the width of the intra-canal fracture fragment was more than 75 % of the transverse diameter of the vertebral canal and the height of intra-canal fracture fragment was more than 47 % of the posterior wall height of the injured vertebrae, the intra-canal fracture fragment could not be reduced by the posterior internal fixation and posterior longitudinal ligament distraction reduction method.
